# Mouse *Nkrp1-Clr* Gene Cluster Sequence and Expression Analyses Reveal Conservation of Tissue-Specific MHC-Independent Immunosurveillance

**DOI:** 10.1371/journal.pone.0050561

**Published:** 2012-12-04

**Authors:** Qiang Zhang, Mir Munir A. Rahim, David S. J. Allan, Megan M. Tu, Simon Belanger, Elias Abou-Samra, Jaehun Ma, Harman S. Sekhon, Todd Fairhead, Haggag S. Zein, James R. Carlyle, Stephen K. Anderson, Andrew P. Makrigiannis

**Affiliations:** 1 Department of Biochemistry, Microbiology, and Immunology, University of Ottawa, Ottawa, Ontario, Canada; 2 Department of Immunology, University of Toronto, Sunnybrook Research Institute, Toronto, Ontario, Canada; 3 Department of Pathology and Laboratory Medicine, The Ottawa Hospital, Ottawa, Ontario, Canada; 4 Kidney Research Centre, Ottawa Hospital Research Institute, Ottawa, Ontario, Canada; 5 Department of Genetics, Cairo University, Giza, Egypt; 6 Basic Science Program, SAIC-Frederick Inc., Laboratory of Experimental Immunology, Frederick National Laboratory for Cancer Research, Frederick, Maryland, United States of America; National Jewish Health and University of Colorado School of Medicine, United States of America

## Abstract

The *Nkrp1* (*Klrb1*)-*Clr* (*Clec2*) genes encode a receptor-ligand system utilized by NK cells as an MHC-independent immunosurveillance strategy for innate immune responses. The related Ly49 family of MHC-I receptors displays extreme allelic polymorphism and haplotype plasticity. In contrast, previous BAC-mapping and aCGH studies in the mouse suggest the neighboring and related *Nkrp1-Clr* cluster is evolutionarily stable. To definitively compare the relative evolutionary rate of *Nkrp1-Clr* vs. *Ly49* gene clusters, the *Nkrp1-Clr* gene clusters from two *Ly49* haplotype-disparate inbred mouse strains, BALB/c and 129S6, were sequenced. Both *Nkrp1-Clr* gene cluster sequences are highly similar to the C57BL/6 reference sequence, displaying the same gene numbers and order, complete pseudogenes, and gene fragments. The *Nkrp1-Clr* clusters contain a strikingly dissimilar proportion of repetitive elements compared to the *Ly49* clusters, suggesting that certain elements may be partly responsible for the highly disparate *Ly49* vs. *Nkrp1* evolutionary rate. Focused allelic polymorphisms were found within the *Nkrp1b/d* (*Klrb1b*), *Nkrp1c* (*Klrb1c*), and *Clr-c* (*Clec2f*) genes, suggestive of possible immune selection. Cell-type specific transcription of *Nkrp1-Clr* genes in a large panel of tissues/organs was determined. *Clr-b* (*Clec2d*) and *Clr-g* (*Clec2i)* showed wide expression, while other *Clr* genes showed more tissue-specific expression patterns. *In situ* hybridization revealed specific expression of various members of the *Clr* family in leukocytes/hematopoietic cells of immune organs, various tissue-restricted epithelial cells (including intestinal, kidney tubular, lung, and corneal progenitor epithelial cells), as well as myocytes. In summary, the *Nkrp1-Clr* gene cluster appears to evolve more slowly relative to the related *Ly49* cluster, and likely regulates innate immunosurveillance in a tissue-specific manner.

## Introduction

Natural killer cells are lymphocytes that are intimately involved in innate host resistance to pathogens and cancer. They patrol the host and survey for the presence of cell surface markers that distinguish healthy versus diseased cells through a variety of receptors [1]. The best characterized examples of receptors include members of the Ly49 family, which bind to class I MHC (MHC-I) and inhibit NK cell responses, and the NKG2D receptor, which binds to MHC-related ligands that are induced following genotoxic or infectious stress, resulting in the activation of NK cells, in turn leading to target cell death and/or induction of cytokine production [2]. The NKR-P1 family of receptors is evolutionarily related to the Ly49 family, but does not survey MHC-I ligands, instead binding to various members of the C-type lectin-related (Clr, also called Ocil) family [3]. Interestingly, the *Nkrp1 (Klrb1)* and *Clr* (*Clec2*) gene families are closely linked, and are in fact intermingled in the Natural Killer gene Complex (NKC) [4].

The known expressed NKR-P1 receptors in the mouse include NKR-P1A (encoded by *Klrb1a*), NKR-P1B (*Klrb1b*), NKR-P1C (*Klrb1c*), NKR-P1F (*Klrb1f*), and NKR-P1G (*Klrb1g*) [5], [6]. NKR-P1D (*Klrb1b*) likely represents an allelic (B6) form of NKR-P1B [7], and *Nkrp1e (Klrb1-ps1)* is a pseudogene in all mouse strains studied to date (see Table 1). The known Clr ligands include Clr-a (encoded by *Clec2e*), Clr-b (*Clec2d*), Clr-c (*Clec2f*), Clr-d (*Clec2g*), Clr-f (*Clec2h*), and Clr-g (*Clec2i*). Clr-e (a.k.a., *Clec2d5*) is a pseudogene [8], while the functional status of Clr-h (*Clec2j*) and Clr-i (no official gene name) are unknown, although Clr-h potentially encodes for a full-length protein in the B6 mouse strain [6]. The available mAb for the mouse NKR-P1 receptors (B6 strain) have revealed that NKR-P1A, NKR-P1B(D), NKR-P1C, NKR-P1F are all expressed on NK cells, but with interesting variation [9]–[11]. Specifically, the putative activating, ITIM-lacking NKR-P1A and NKR-P1F surface proteins are expressed at low levels on all NK cells, but not NKT cells [9]. However, it should be noted that neither IFN-γ nor redirected lysis could be induced with mAb to NKR-P1A or NKR-P1F [9], unlike NKR-P1C [12]. In contrast to NKR-P1A and NKR-P1F, the activating NKR-P1C^B6^ (NK1.1^B6^) is expressed at high levels on all NK and NKT cells, while NKR-P1B is stochastically expressed (similar to the Ly49) on approximately 60% of NK cells [9], [10]. At present, mAb for the Clr family are not commercially available, but mAb recognizing Clr-b show that it is broadly expressed on most leukocytes [13], and is down-regulated in response to genotoxic stress and viral infection [14], [15], suggesting it acts as a marker of ‘health’, similar to MHC-I, to inhibit NK cell responses. The known receptor-ligand pairs have been identified through colorimetric reporter cell assays, receptor down-regulation assays, and in some cases through NK cell cytotoxicity assays. These binding pairs include: NKR-P1B/D:Clr-b, NKR-P1F:Clr-c,d,g and NKR-P1G:Clr-f,d,g [6], [10], [13], [16]. In the rat, among 4 *Nkrp1* and 11 *Clr* genes, the known interactions include NKR-P1A:Clr11, NKR-P1B:Clr11, NKR-P1F:Clr2,-3,-4,-6,-7, and NKR-P1G:Clr2,-6,-7 and weak binding to Clr-4 [16]. In humans, there is a single gene encoding NKR-P1A (*KLRB1/CD161*), which shows inhibitory function upon binding the Clr-related ligand LLT1 (*CLEC2D*) [17], [18]. However, KLRF1 (NKp80) and KLRF2 (NKp65) have been suggested recently to represent possible stimulatory NKR-P1/KLRB1 homologs, and they bind to the genetically linked ligands, CLEC2B (AICL) and CLEC2A (KACL), respectively [19].

**Table 1:** *Nkrp1/Clr* gene nomenclature.

Common Name(s)	Official Gene Symbol (or Suggested Name)
*Nkrp1a*	*Klrb1a*
*Nkrp1b*	*Klrb1b*
*Nkrp1c*	*Klrb1c*
*Nkrp1d*	*Klrb1b*
*Nkrp1e*	*Klrb1-ps1*
*Nkrp1f*	*Klrb1f*
*Nkrp1g*	*Klrb1 (Klrb1g)*
*Clr-a (Clec2d7)*	*Clec2e*
*Clr-b (Ocil/Clec2d8)*	*Clec2d*
*Clr-c (Clec2d6)*	*Clec2f*
*Clr-d (Ocilrp1/Ddv10//Clec2d4)*	*Clec2g*
*Clr-e (Clec2d5)*	*– (Clec2-ps1)*
*Clr-f (Clec2d2)*	*Clec2h*
*Clr-g (Ocilrp2/Dcl1/LCL-1/Clec2d3)*	*Clec2i*
*Clr-h (Clec2d1)*	*Clec2j*
*Clr-i*	*– (Clec2-ps2)*
*Clr-j*	*– (Clec2-ps3)*

Although related, there are additional notable differences between the Ly49 and NKR-P1 receptor families, in addition to their unrelated ligands. Firstly, while the majority of Ly49 contain ITIM motifs, this is not the case for the NKR-P1 family of receptors. NKR-P1B(D) contain an ITIM and inhibit NK cell activation upon binding to the ligand, Clr-b [20]; and a second ITIM-containing NKR-P1, NKR-P1G, has been identified at the gene and cDNA level [6], but its cell-type expression and function remain undocumented to date. The remaining NKR-P1 receptors (NKR-P1A/C/F) do not contain ITIM motifs, but instead possess a positively charged residue in the transmembrane domain, presumably for adaptor molecule association (as has been shown for NKR-P1C and FcRγ [21]), and variably contain other features (such as single tyrosine residues, CxCP Lck-recruitment motifs, and proline-rich regions [22], [23]). Secondly, the *Ly49* gene clusters of various inbred mouse strains are highly polymorphic at the level of allelic diversity and in terms of gene numbers, with known haplotypes ranging from 8–22 genes [3]. In contrast, the *Nkrp1-Clr* haplotypes appear to be highly conserved with little or no variation in gene organization or content, and only focused allelic diversity [3]. However, only a single *Nkrp1-Clr* cluster sequence, from B6 mice, has so far been available; nonetheless, BAC-based gene maps for the 129S6 and BALB/c mouse strains have been constructed [6], [7]. In addition, recent array-based comparative genomic hybridization (aCGH) studies support the contention that the *Nkrp1-Clr* region shows less inbred mouse-strain variability than the *Ly49* region [24]. However, definitive multi-strain genomic sequence data is lacking for this gene cluster.

To further characterize the genetic organization and function of the NKR-P1-Clr self-recognition system utilized by NK cells, we report here the high-resolution sequencing of two *Nkrp1-Clr* haplotypes from 129S6 and BALB/c mice, and confirm the relative stability of this region of the NKC, including the characterization of novel genes. In order to provide insight into the functional roles of different NKR-P1-Clr receptor-ligand pairs, RT-PCR was performed for all *Nkrp1-Clr* genes in a large panel of tissues and organs. Additionally, the lack of specific commercially available mAb for the Clr family prompted us to use *in situ* hybridization of various organs to identify specific cell-types expressing *Clr* transcripts. We report here the expression of various Clr members in the epithelial cells lining several organs and other non-hematopoietic cell types, supporting a novel function for NK cells in tissue immunosurveillance.

## Materials and Methods

### Mice

C57BL6/J, BALB/c, and 129S1 mice were obtained from The Jackson Laboratory (Bar Harbor, ME), and maintained in a specific-pathogen-free environment. All breeding and manipulations performed on animals were in accordance with university guidelines and approved by the University of Ottawa Animal Ethics Committee.

### BAC Sequencing and Analyses

BACs were sequenced to 20–30x coverage using a Roche 454 GS FLX Titanium 2 instrument at Genome Quebec Innovation Centre (Montreal, QC) and The Centre for Applied Genomics, SickKids Hospital (Toronto, ON). The final cluster sequence was assembled using GS data analysis software (Roche). Gaps in the assembled sequence are primarily due to the presence of low complexity regions of lengths that could not be precisely defined, therefore gaps were introduced to indicate this uncertainty. Ordering of contigs was based on the order of known coding regions that were confirmed from cDNA clones obtained from the identical mouse strain. Final assemblies of the BALB/c and 129S6 strains were compared to each other and the complete B6 sequence to confirm that there were no assembly errors. The following BACs from the 129S6 genomic library RPCI22 were sequenced: 570e7, 525h12, 381m24, 519p18, 410a1, 475e14, and 439c16. The accession number for the BAC sequences covering the 129S6 *Nkrp1-Clr* cluster is JQ948150. For the BALB/c *Nkrp1-Clr* region, the following BACs from library CHORI-28 were sequenced: 387p19, 358o7, 451l24, 371k12, and 445a21. The accession number for the BAC sequences covering the BALB/c *Nkrp1-Clr* cluster is JX026279. For gene sequence comparison, the ‘align two or more sequences’ feature of Blastn was used to align the region of interest from the first to last exon as annotated for each gene. The assembled cluster sequences were analyzed for unknown *Nkrp1*- and *Clr*-related genes by taking 10–20 nucleotide segments of the known exons and searching the genomic sequence using Lasergene sequence analysis software (DNASTAR Inc., Madison WI). Repetitive elements were identified with RepeatMasker version 3.1.8 (A.F.A. Smit and P. Green unpublished; available at http://www.repeatmasker.org). A PIP of the repeat-masked *Nkrp1-Clr* cluster sequences from the various inbred mouse strains (versus the B6 sequence) was constructed using Advanced Pipmaker with a setting of single coverage (available at http://pipmaker.bx.psu.edu/pipmaker).

### Tissue RT-PCR

Adult B6, 129S1 or BALB/c mice were anesthetized with avertin, perfused transcardially with PBS and different tissues were collected. Tissues were rinsed with PBS, frozen with liquid nitrogen and transferred to −80° before RNA isolation. RNA was isolated from tissues using RiboZol RNA extraction reagent (AMRESCO). RNA (1 µg) was reverse-transcribed into cDNA using Verso cDNA kit (Thermo Scientific). PCR amplification was performed on 1 µl of the above cDNA product using primers specific for *Nkrp1-Clr* and *Gapdh* genes (Table 2). PCR products were visualized on a 1% agarose gel stained with ethidium bromide. Primer specificity was checked using serial dilution of plasmids (1 ng, 10^−1 ^ng, 10^−2 ^ng, 10^−3 ^ng) containing different *Nkrp1* and *Clr* cDNAs as template for PCR amplification. Previous microarray expression studies done for the mouse are available online (http://biogps.org) [25], [26], and were used to confirm RT-PCR results.

**Table 2:** Primers used to amplify *Nkrp1- Clr* genes from tissue cDNA and plasmids.

Primer specificity	Sense primer	Antisense primer
*Clr-a*	CAAAGGTTGAAGAGGCTTCC	TCACGCATGCTTTGGCACAT
*Clr-b*	ACTCAGCTCCTCAGCTCTGA	GGCTAAAAAGCGTCTCTTGG
*Clr-c*	GTTATGACAGCCTCACAGGA	GCTAGCACTGTAACATATAG
*Clr-d*	GTTATGAATGCACAGTGCCT	GCTAGACAGGAACAGGAGTT
*Clr-f*	TTGAAACGAGTTCCATGGGC	GGTCATAGAGCATCTGATTG
*Clr-g*	AGATTGCTTGGAGACAGGAG	GAAGAGTCTCTTGGTAAGTG
*Clr-h*	GTTATGCAGATGAAGGCGAA	GCTAGCACAGTAAGGTGTAG
*Nkrp1a*	TGATGCATCTCCTATGCACA	CTCTTGGACAGGATCTGAGT
*Nkrp1d^B6^*	GGTGTCAAGTCCCTCCATCT	CATCAGAATTGAAAGCTGTG
*Nkrp1b^129&BALB^*	ATGGATTCAACAACACTGGT	TCAGGAGTCATTACACGGGG
*Nkrp1c*	CAGTGGATCCCCATCAAGAGAAAAATGCTG	ACTGGAATTCTCAGGAGTCATTACTTGGGG
*Nkrp1f*	GTTCCCATCTCCTGTCTACA	AGGAATCAGGACACAGGCTT
*Nkrp1g*	ATGGATGCACCAGTGCTCTA	TCAGACGTGTTTCAGTGTCT
*Gapdh*	ACTCACGGCAAATTCAACGGC	ATCACAAACATGGGGGCATCG

### *In situ* Hybridization

*In situ* hybridization analysis for *Clr* genes in different tissues was performed as described previously [27]. Digoxigenin-labeled antisense and sense RNA probes were transcribed from linearized plasmids containing *Clr* genes with T7 polymerase using DIG RNA labeling mix (Roche). Tissues were collected from adult B6 mice and were fixed either in 10% formalin at RT or in 4% paraformaldehyde at 4° with gentle shaking for up to 24 h. Tissues were then embedded in paraffin and 4 µm sections were prepared on glass slides. Tissue slides were deparaffinated, treated with 0.2 N HCl for 15 min and 30 µg/ml proteinase K at 37° for 20 min, fixed in 4% PFA for 10 min and incubated with 0.25% acetic anhydride in 0.1 M triethanolamine twice for 5 min each. Slides were then pre-hybridized with hybridization solution (50% (v/v) formamide/5× SSC, pH 4.5; 2% (w/v) blocking powder (Roche); 0.05% (w/v) CHAPS; 5 mM EDTA; 50 µg/ml heparin; 1 µg/ml yeast RNA) in a 58° oven for at least 1 h, and then incubated with hybridization solution containing 500 ng/ml digoxigenin-labeled probe overnight at 58°. Post-hybridization washes were performed with 50% formamide/2X SSC, pH 4.5 at 55° 3x for 20 min each and slides were stained with sheep anti-digoxigenin alkaline phosphatase-conjugated antibody at 1/1000 dilution in blocking solution (Roche). Color development was performed by adding nitro blue tetrazolium/5-bromo, 4-chloro, 3-indoylphosphate (NBT/BCIP, Roche) substrates to the slides and slides were kept in the dark at RT until optimal signals appeared on slides. Slides were then counterstained with lightgreen or methylgreen and visualized under a light microscope.

## Results

### Organization and Content of the B6 *Nkrp1-Clr* Gene Cluster

**Figure 1:**
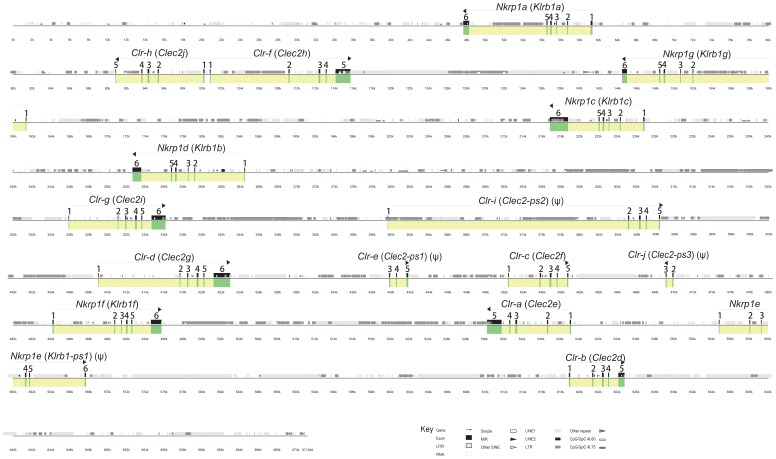
A graphical representation of the organization of the *Nkrp1-Clr* gene cluster in the C57BL/6 (B6) inbred mouse strain. The UCSC Genome Browser was used to capture the genomic sequence between and including the *Nkrp1a* and *Clr-b* genes. Genes were identified by Lasergene, using short homology searches of B6 *Nkrp1/Clr* cDNAs. The locations of various kinds of repetitive elements were revealed with RepeatMasker and are shown (see key). Advanced Pipmaker was used to construct a scale diagram of the exons and a PIP vs. itself (single coverage). Genes and exons are marked in yellow and green, respectively. The sequence is demarcated below the plot in kilobases. Arrows show gene orientation, and the common gene name followed by the official gene name (in parentheses) or suggested official name are indicated above the arrow. Obvious pseudogenes are denoted with a ψ.

The *Nkrp1-Clr* (*Klrb1-Clec2*) cluster of B6 mice has been characterized previously in moderate detail [4], [5], [24]. To compare the *Nkrp1-Clr* gene cluster sequences in the B6, 129S6, and BALB/c strains, the reference B6 *Nkrp1-Clr* region was fully assembled using publicly available genomic data (**Figure 1**). The relative order of genes in the B6 genome is: *Nkrp1a* (*Klrb1a*), *Clr-h* (*Clec2j*), *Clr-f* (*Clec2h*), *Nkrp1g* (*Klrb1g*), *Nkrp1c* (*Klrb1c*), *Nkrp1b/d* (*Klrb1b*), *Clr-g* (*Clec2i*), *Clr-i* (*Clec2-ps2*), *Clr-d* (*Clec2g*), *Clr-e* (*Clec2-ps1*), *Clr-c* (*Clec2f*), *Clr-j* (*Clec2-ps3*), *Nkrp1f* (*Klrb1f*), *Clr-a* (*Clec2e*), *Nkrp1e* (*Klrb1-ps1*), and *Clr-b* (*Clec2d*) [6]–[8]. The cluster is approximately 580 kb in length and has a gene order and content consistent with the previously published 129S6 and BALB/c BAC-based maps, including a new gene fragment we term *Clr-j* (*Clec2-ps3*) [6], [7]. Similar to the *Ly49* (*Klra*) genes, the protein coding regions of the *Nkrp1* genes are divided over 6 exons. In contrast, the *Clr* genes usually contain 5 coding exons, except for *Clr-d* (*Clec2g*) and *Clr-g* (*Clec2i*), which have 6 exons due to the presence of an additional 5′ coding exon.

Sequence database searches for *Nkrp1-Clr*-related exons in the B6 genome using 10–20 nucleotide segments of all known B6 *Nkrp1*- and *Clr*-related cDNAs facilitated the discovery of a novel gene fragment consisting of two exons (exons-2 and -3, relative to other complete 6-exon *Clr* genes), which we have termed *Clr-j* (tentatively *Clec2-ps3*). Another obvious *Clr* pseudogene is *Clr-i* (*Clec2-ps2*), which we identified previously from two exon fragments in 129S6 mice [6]. We have found that the *Clr-i* gene in B6 mice contains all necessary exons but has multiple in-frame stop codons in all three reading frames (data not shown). *Clr-e* (*Clec2-ps1*) is also a pseudogene, as only three exons (exons-3, -4 and -5) have been identified. In contrast, only a single *Nkrp1* gene, *Nkrp1e* (*Klrb1-ps1*), is a pseudogene in B6 mice, as it contains multiple early stop codons (data not shown). It is noteworthy that the *Clr-h* (*Clec2j*) and *Clr-f* (*Clec2h*) genes are arranged in a head-to-head orientation and their respective first exons are less than 1 kb apart. When the *Clr-f* cDNA was used to query the EST database, multiple *Clr-f* clones from different tissues were discovered: AF350410.1, NM_053165.5, AK090364.1, BC021766.1, AK017207.1, HQ875061.1, HQ713437.1. However, all these cDNAs contained exon-1 (data not shown), suggesting that the promoter for *Clr-f* is either in the 1 kb region separating the *Clr-f* and *Clr-h* genes or may include part of the *Clr-h* gene. The functional status of the *Clr-h* gene is unknown; no transcripts have been reported in the EST database or otherwise in the literature, although a complete *Clr-h* open reading frame is present in B6 mice.

Sequencing of the related *Ly49* gene cluster in several inbred strains of mice has revealed an overrepresentation of repetitive elements [28], [29]. Specifically, the LINE1 family of retroelements represents ∼36% of the *Ly49* region in all of the four distinct sequenced haplotypes (B6, BALB/c, 129, NOD), which range in size from 8–22 genes. In contrast, the mouse genome contains ∼19% LINE1 elements. We have hypothesized that intra-cluster meiotic recombination due to LINE1 overrepresentation helps to drive *Ly49* evolution [3]. The relative conservation of the neighboring and related *Nkrp1-Clr* gene cluster provides an opportunity to test this hypothesis. The presence of various types of repeats in the B6 *Nkrp1* versus *Ly49* region is summarized in **Table 3**. While the overall percentage of interspersed repeats is similar in the two gene regions (∼54% vs. ∼51%), there are two striking differences: 1) the *Nkrp1-Clr* cluster consists of only ∼22% (vs. ∼36% in the *Ly49* clusters) LINE1 sequences, which is similar to the total genome average, and 2) there are more than twice as many LTR elements in the *Nkrp1-Clr* region as in the *Ly49* region (∼26% vs. ∼12%). The first difference supports the hypothesis that LINE1 elements may drive unequal crossing-over between sister chromatids, but the implications for LTR overrepresentation in the *Nkrp1-Clr* region are unclear.

**Table 3:** Comparison of repeats in the *Nkrp1-Clr* and *Ly49* gene clusters from B6 mice.^a^

*Type of element*	*Number of elements*		*Length occupied (bp)*		*Percentage of sequence*	
	Nkrp1	Ly49	Nkrp1	Ly49	Nkrp1	Ly49
***SINES***						
Alu/B1	94	99	11303	11148	1.68	1.95
B2–B4	106	35	15205	4936	2.27	0.86
IDs	12	5	832	376	0.12	0.07
MIRs	9	0	1207	0	0.18	0.00
Total	221	139	28547	16460	4.25	2.87
***LINEs***						
LINE1	148	264	147150	203094	21.92	35.46
LINE2	9	4	1133	348	0.17	0.06
L3/CR1	1	0	46	0	0.01	0.00
Total	158	268	148329	203442	22.10	35.52
***LTR elements***						
ERVL	21	2	5500	791	0.82	0.14
ERVL-MaLRs	57	26	17394	12857	2.59	2.24
ERV_classI	38	18	18508	14752	2.76	2.58
ERV_classII	182	43	136954	40436	20.40	7.06
Total	298	89	178356	68836	26.57	12.02
**DNA elements**						
hAT-Charlie	6	4	902	305	0.13	0.05
TcMar-Tigger	17	0	3784	0	0.56	0.00
Total	25	4	4939	305	0.74	0.05
**Unclassified**	2	1	1866	45	0.28	0.01
Total interspersed repeats			362037	289088	53.93	50.48
**Small RNA**	14	8	1028	513	0.15	0.09
**Satellites**	3	2	313	197	0.05	0.03
**Simple repeats**	186	199	10491	12840	1.56	2.24
**Low complexity**	64	110	3236	5281	0.48	0.92

a As detected by RepeatMasker.

### Sequence Analysis of the 129S6 and BALB/c *Nkrp1-Clr* Gene Clusters

**Figure 2:**
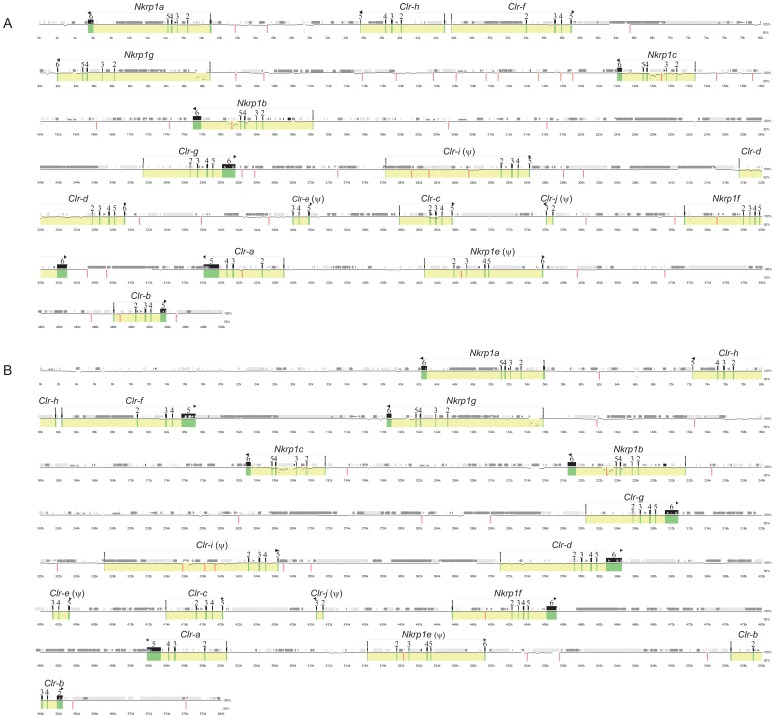
A graphical representation of the organization of the *Nkrp1-Clr* gene cluster in (A) 129S6 and (B) BALB/c inbred mouse strains. A minimal BAC tiling path was selected from previous BAC mapping reports and next-generation sequencing was performed. Contigs were assembled using GS data analysis software. Genes were identified by Lasergene, using 10–20 nucleotide sequences of *Nkrp1/Clr* cDNAs from 129S6 or BALB/c mice. Advanced Pipmaker was used to construct a scale diagram of the exons and a PIP vs. the B6 *Nkrp1-Clr* cluster sequence (single coverage). Genes and exons are marked in yellow and green, respectively. The sequence is demarcated below the plot in kilobases. Arrows show gene orientation, and the common gene name is above the arrow. Obvious pseudogenes are denoted with a ψ. The locations of various kinds of repetitive elements were revealed with RepeatMasker and are shown (see key in Figure 1). Vertical red bars indicate gaps between contigs. Accession numbers for the cluster sequences are given in the Materials and Methods.

In agreement with our *Ly49* and *Nkrp1-Clr* haplotype studies, a recent report utilized aCGH analyses of a large panel of inbred mouse strains in support of the hypothesis that the *Nkrp1-Clr* cluster is less diverse compared to the *Ly49* region [24]. As discussed above, the 129S6 and BALB/c *Nkrp1-Clr* genes have been mapped using BAC clones [6], [7]. The organization, number, and type of genes appear to be identical to the B6 cluster, as shown in **Figure 1**; however, only high resolution sequencing can definitively reveal the degree of relatedness among all three clusters and any potential hidden genes. Therefore, a minimal BAC tiling assembly was chosen for both the 129S6 and BALB/c clusters and sequenced. The exon organization of the BALB/c and 129S6 *Nkrp1-Clr* regions is shown in **Figure 2**. The *Nkrp1* and *Clr* genes in both the 129S6 and BALB/c haplotypes were identical to the B6 haplotype in terms of gene content (*Nkrp1a*-*Nkrp1g* and *Clr-a-Clr-j*), including all functional genes, as well as complete and fragmentary pseudogenes (**Figure 2**).

**Figure 3:**
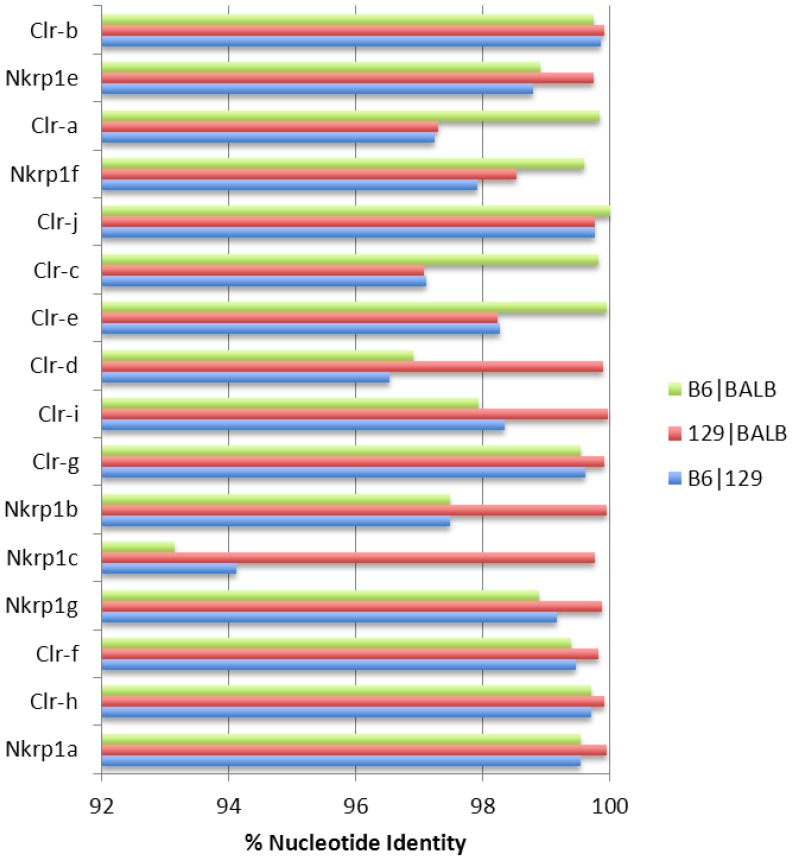
Comparisons of *Clr* and *Nkrp1* gene homologies between three inbred mouse strains. The genomic region of each gene from the translational start codon to the termination codon was subjected to pair-wise comparison (B6/BALB; 129/BALB; B6/129) using Blastn. The percent nucleotide identity of each gene for the three strain pairs is shown.

The three haplotypes are highly related and share ≥98% sequence identity overall (**Figure 3**), but with some gene-specific differences. For example, for the *Nkrp1a* gene, the 129S6 allele is 99.54% identical to the B6 allele, whereas the BALB/c allele is 99.65% identical to the B6 allele, and the 129S6 and BALB/c alleles are 99.96% identical to each other. In fact, from *Nkrp1a* to *Clr-d*, 129S6 and BALB/c are >99.5% identical and B6 identity to either 129S6 or BALB/c is more variable (**Figure 3**). In particular, BALB/c and B6 *Nkrp1c* alleles are ∼93% identical, while the BALB/c and B6 *Nkrp1b/d* alleles are ∼97% identical. In direct contrast, the BALB/c segment encompassing *Clr-e*, *Clr-c*, *Clr-j*, *Nkrp1f*, and *Clr-a* is highly homologous (>99.5%) to the corresponding B6 segment, suggesting a common origin, possibly as a result of a double cross-over event resulting in intra-cluster recombination.

Previous studies have identified cloned *Nkrp1-Clr* cDNA sequences from various mouse strains [6]; however, some cDNAs from intact genes remain unreported, possibly due to restricted or unknown tissue-specific expression. Therefore, the putative exonic sequences of all BALB/c and 129S6 *Nkrp1* and *Clr* genes were collected and assembled from the regional sequencing, and were used to ascertain the degree of polymorphism between the cloned and uncloned alleles of all three mouse strains (**Table 4**). We used these data to confirm, at the genomic level, the sequences of the cloned cDNAs of all transcribed *Nkrp1* and *Clr* genes in both mouse strains, including the *Clr-c* alleles, which we previously observed were highly divergent (38/206 aa differences) between the 129-strain allele compared to the BALB/c and B6 Clr-c alleles, which are identical [6]. These data are thus far also consistent with the 129S1 and 129S6 substrains having identical *Nkrp1*, *Clr*, and *Ly49* gene sequences, as the isolated cDNA (129S1 strain) and genomic DNA templates (129S6 strain) were identical to each other. Among the three inbred strains, the degrees of relatedness for most alleles were ∼99% or greater (nucleotide or aa), except for the previously noted divergence of the *Clr-c*, *Nkrp1b/d*, and *Nkrp1c* alleles.

**Table 4:** *Nkrp1* and *Clr* homology in 129S6, B6, and BALB/c mice.

	cDNA^a^			amino acid		
	129 vs B6	BALB vs B6	129 vs BALB	129 vs B6	BALB vs B6	129 vs BALB
*Clr-a*	98%	99%	99%	97%	99%	97%
*Clr-b*	100%	100%	100%	100%	100%	100%
*Clr-c*	92%	100%	92%	82%	100%	82%
*Clr-d*	98%	98%	100%	97%	97%	100%
*Clr-f*	99%	99%	100%	99%	99%	100%
*Clr-g*	100%	100%	100%	100%	100%	100%
*Clr-h*	100%	100%	100%	100%	100%	100%
*Nkrp1a*	99%	99%	100%	99%	99%	100%
*Nkrp1b(d)*	96%	96%	100%	90%	90%	100%
*Nkrp1c*	94%	94%	100%	88%	88%	100%
*Nkrp1f*	99%	100%	99%	99%	100%	99%
*Nkrp1g*	99%	99%	100%	99%	99%	100%

a cDNAs were compared from the translational start to stop codons to ascertain sequence identity.

Interestingly, we found that the fragmentary pseudogenes *Clr-e* (*Clec2-ps1*) and *Clr-j* (*Clec2-ps3*) and the complete pseudogene *Clr-i* (*Clec2-ps2*) were present in all three haplotypes with the same number of exons (*Clr-e*: exons-3, -4, and -5; *Clr-i*: exons-1-5; *Clr-j*: exons-2 and -3), which is somewhat surprising, as it is expected that there would be no selective pressure to retain these non-coding genes. As previously noted, the *Clr-h* gene appears to be intact, as there are no premature stop codons in the open reading frame, but it might represent a pseudogene, as no transcript was detectable by RT-PCR in NK cells. The closest EST database match for *Clr-h* is *Clr-b* (∼95% identity). However, *Clr-h* is 100% conserved in exonic nucleotide sequence among all three strains (**Table 4**), which would be remarkable for a non-functional gene, and strongly suggests that a candidate cell-type that expresses Clr-h has not yet been identified.

**Figure 4:**
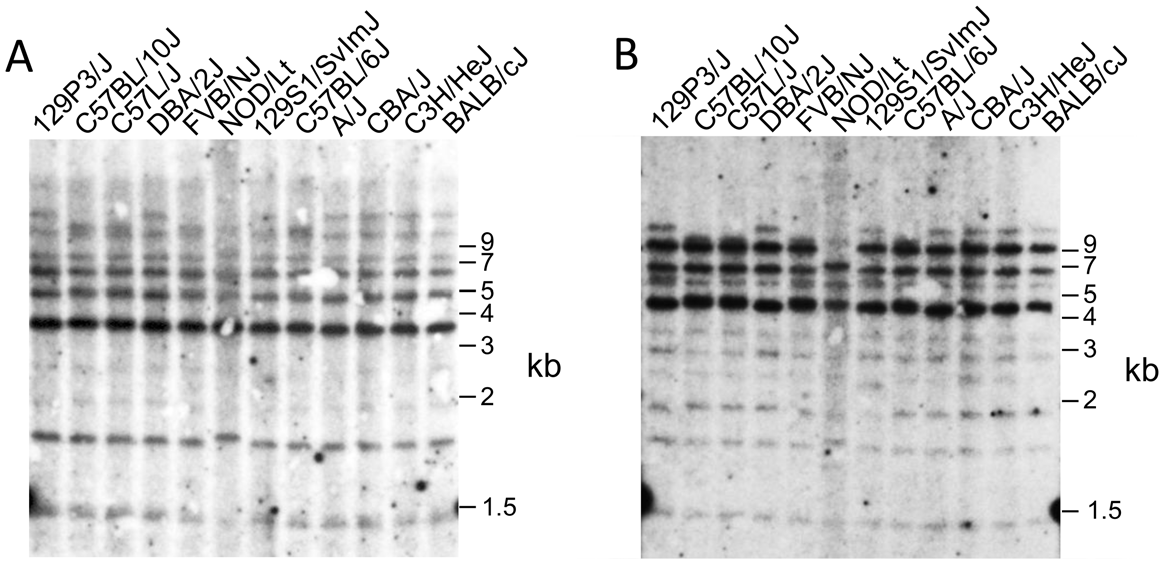
Evolutionary conservation of *Clr* genes in the mouse. Thymic genomic DNA from the indicated inbred mouse strains was digested with EcoRI and subjected to southern blot analyses using ^32^P-labelled cDNA probes for (A) *Clr-b*, and (B) *Clr-f*. The membrane was stripped between hybridizations.

In line with our BAC sequencing results and previous studies [7], Southern blot analyses of genomic DNA from a panel of inbred mouse strains, using the *Clr-b* and *Clr-f* cDNA sequences as probes, indicated that these *Clr* gene RFLP patterns are highly similar. In turn, these results suggest that not only are the *Clr-b* and *Clr-f* genes well conserved, but in addition, due to cross-hybridization, that most if not all *Clr* genes in general are quite conserved, although some polymorphisms are present (**Figure 4**). Finally, the location and frequency of repetitive elements in the three *Nkrp1-Clr* haplotypes was found to be similar (**Figures 1** **,** **2** **and** **Table 3**). In summary, in agreement with our previous BAC mapping and cDNA cloning studies [6], [7], as well as aCGH studies from another group [24], the *Nkrp1-Clr* gene cluster appears to be quite conserved and evolutionarily more stable relative to the *Ly49* cluster; nonetheless, specific genes appear to be evolving more rapidly than others, and like the *Ly49* region [3], [30], there is evidence of recombination between distinct *Nkrp1-Clr* haplotypes.

### Characterization of *Nkrp1-Clr* Transcript Expression in Tissues and Organs

The genomic sequencing of the *Nkrp1-Clr* region has facilitated the identification and mapping of conserved genes and allelic/regional polymorphisms, including exonic and intronic sequences for the three inbred strains. Applying these findings, the specific tissues and cell types expressing *Nkrp1-Clr* genes were analyzed next. Tissue-specific, and, in some cases, cell-type-specific micro-array analyses have been performed previously to characterize global transcript expression of mouse genes, including most *Nkrp1* and *Clr* genes, and these findings are publicly available on the BioGPS website [26]. To independently confirm and extend previous tissue expression studies [4], the transcript expression of all functional *Nkrp1* and *Clr* genes in a panel of tissues and organs from the three inbred strains was analyzed by RT-PCR.

**Figure 5:**
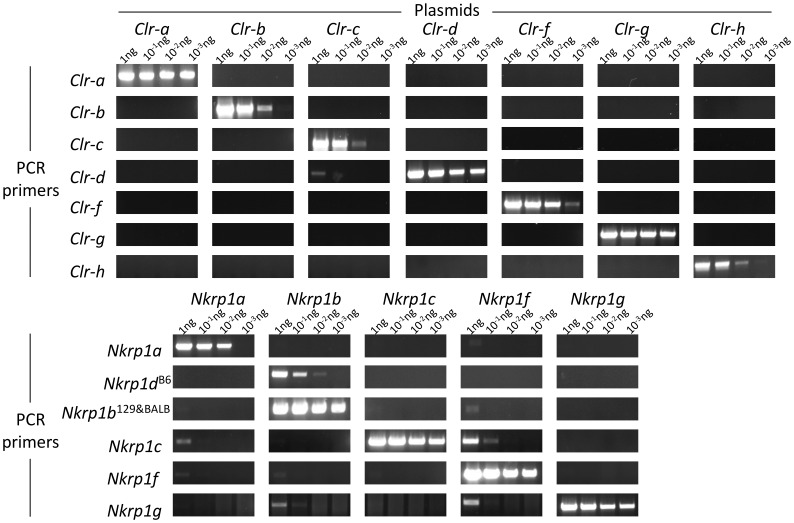
Testing of *Nkrp1-Clr* primers for PCR specificity. Cloned cDNAs for each of the known *Nkrp1* and *Clr* genes were used as a template at the indicated amounts in PCR amplification experiments with primers for each of the genes. The resultant DNA amplification product is shown after agarose gel electrophoresis. Primer specificity is indicated on the left and plasmid template across the top.

First, each set of gene-specific primers was tested for specificity against cloned cDNAs by serial dilution of template plasmid DNA. Overall, it was determined that the primers utilized were quite specific for the particular genes they were originally designed to amplify (**Figure 5**). Minor cross-amplification was observed for select primer sets, including faint detection of *Clr-c* cDNA (by *Clr-d* primers), *Nkrp1a* cDNA (by *Nkrp1c* primers), *Nkrp1b/d* cDNA (by *Nkrp1g* primers), and *Nkrp1f* cDNA (by *Nkrp1a,b*,*c,g* primers); however, this was typically observed only at the highest amounts of template plasmid DNA (1 ng), and is thus unlikely to be a confounding factor for *ex vivo* tissue-specific RT-PCR analysis, where typically ∼50 ng or less of total cDNA is used per reaction.

Next, RT-PCR for each *Nkrp1* and *Clr* gene product was performed on RNA isolated from 15 tissues and organs: tongue, muscle (hind leg), liver, bladder, spleen, thymus, ovaries, testes, eye, kidney, heart, lung, lymph node, brain, and intestine. The analyses were performed for the 129S1, B6, and BALB/c strains, and the expression results are shown in **Table 5** and **Table 6** **.** The corresponding micro-array results, as previously reported [25], [26], are shown underneath the RT-PCR results for each gene/tissue, but micro-array data was not available for *Clr-c*, *Clr-h*, or *Nkrp1g*. Overall, the RT-PCR results agreed with the micro-array data, as well as previous expression studies for the *Nkrp1-Clr* genes [4].

**Table 5:** Tissue and organ expression of *Clr* genes as determined by RT-PCR.

	tongue	muscle	liver	bladder	spleen	thymus	ovaries/testes	eye	kidney	heart	lung	lymph node	brain	intestine
***Clr-a***														
B6	-a	–	–	–	–	–	–	–	–	–	–	–	–	+
BALB	-	–	–	–	–	–	–	–	–	–	–	–	–	+
microarray^b^	-	–	–	–	–	–	–	–	–	–	–	–	–	+
***Clr-b***														
B6	+	+++	++	+	++	++	+	+	+	+	+	++	–	+
129	+	+++	+++	+	+	+	+	−/+	++	++	++	++	–	+
BALB	+	+++	+++	+	++	++	+	−/+	++	+++	++	++	–	+
microarray	+	+	+	+	+	+	+	-	+	+	+	+	–	+
Clr-c														
B6	−/+	–	–	−/+	+	+	+	–	–	–	–	−/+	–	+
129	−/+	–	–	−/+	+	+	−/+	–	−/+	–	–	+	–	+
BALB	+	–	–	–	+	+	+	–	−/+	–	+	−/+	-	+
microarray	ND^c^													
Clr-d														
B6	+	–	–	–	−/+	−/+	–	+	–	–	–	–	–	–
129	–	–	–	–	−/+	–	–	+	–	–	–	–	–	–
BALB	−/+	–	−/+	−/+	−/+	−/+	–	+	–	–	–	–	–	–
microarray	–	–	–	–	–	–	–	+	–	–	–	–	–	–
Clr-f														
B6	−/+	−/+	++	–	−/+	−/+	–	–	+++	–	−/+	−/+	–	+++
129	–	–	++	−/+	+	–	–	–	+++	+	–	−/+	−/+	+++
BALB	–	−/+	–	–	–	–	–	–	+++	–	–	−/+	–	+++
microarray	–	+	+	–	–	–	–	–	+	–	–	–	–	+
Clr-g														
B6	–	–	–	–	++	++	–	–	−/+	–	+	++	–	+
129	–	–	−/+	–	++	+++	−/+	–	−/+	–	+	+++	–	+
BALB	–	−/+	−/+	–	++	++	–	–	–	–	+	++	−/+	++
microarray	–	–	–	–	+	+	–	–	–	–	–	+	–	–
Clr-h														
B6	–	–	–	–	–	–	–	–	–	–	–	–	–	–
129	–	–	–	–	–	–	–	–	–	–	–	–	–	–
BALB	–	–	–	–	–	–	–	–	–	–	–	–	–	–
microarray	ND													

a -, no expression; −/+, very weak expression; +, weak to moderate expression; ++, strong expression; +++, very strong expression.

b Microarray data were obtained from the Mouse MOE430 Gene Atlas database at BioGPS portal server.

c ND, not determined.

*Clr-a* expression was found only in the intestine, and only at low levels. *Clr-b*, which is known to be expressed by most nucleated hematopoietic cells [10], [13], was expressed broadly and strongly in almost all tissues except the brain (**Table 5**), and is in agreement with northern blot analysis showing *Clr-b* (*mOcil*) expression in the liver, kidney, gut, spleen, heart, and smooth muscle [31]. The brain was the only organ negative for all *Nkrp1* and *Clr* transcripts, as assessed by both RT-PCR and micro-array (**Table 5** and **Table 6**). Tissues used for our RT-PCR experiments and the micro-array data were isolated from PBS-perfused animals to avoid misleading interpretations from resident blood cells. However, it is possible that the wide expression of *Clr-b* may have been due to tissue-embedded resident leukocytes, which have been previously reported to express *Clr-b* [10]. *Clr-c* was expressed at low levels in the tongue, spleen, thymus, ovaries, testes, and lymph node tissues. *Clr-d* transcripts were present specifically in the eye. *Clr-f* was expressed in the liver and very highly in the kidney and intestine. *Clr-g* was consistently expressed in the spleen, thymus, and lymph node. Our RT-PCR results clearly indicated that *Clr-g* is also expressed in the lung and intestine, but these findings disagreed with micro-array results, which reported no *Clr-g* expression in these tissues. This could be due, in part, to alternate splicing of *Clr-g* gene products [32], in turn detected differentially by distinct primer and probe sets. Finally, while the *Clr-h* gene appears to be complete with an intact coding region, as well as being 100% conserved across all three mouse strains, no expression was detected in any of the tissues analyzed. Thus, most *Clr* genes appear to be expressed in a tissue-specific manner, with some overlap between isoforms and some discrepancies revealed by distinct detection methods. Whether the overall Clr expression patterns are dependent upon non-hematopoietic cell types or tissue-resident leukocytes, which remains possible for Clr-b [10], [13], requires further inquiry.

In contrast, the *Nkrp1* family of genes was more consistent in terms of expression patterns. In general, wherever NK cells (or related lymphocytes) have been reported in abundance, both the RT-PCR and micro-array results were positive (**Table 6**). Transcripts for all *Nkrp1* genes were detected in the spleen, thymus, lung, and lymph node tissues. Additionally, based on our RT-PCR results, the intestine showed expression of almost all *Nkrp1* transcripts, with the exception of *Nkrp1a*. Furthermore, we found the liver to be positive for *Nkrp1b/d*, *Nkrp1c*, and *Nkrp1f* transcripts, although the micro-array results were not always consistent with these RT-PCR results. However, given the well-known abundance of liver-resident NK (and NKT) cells, it is likely that the micro-array results may not have sufficient sensitivity to detect expression in subsets of these cells; alternatively, PBS perfusion may have inadvertently removed these immune populations from the liver tissue. Finally, *Nkrp1b/d* was somewhat unique in showing expression within the tongue and bladder. Like the *Clr* transcripts, no *Nkrp1* transcripts were detected in the brain, in contrast to the related *Ly49* transcripts, which have been reported to be abundantly expressed in neurons [33].

**Table 6:** Tissue and organ expression of *Nkrp1* genes as determined by RT-PCR.

	tongue			muscle		liver		bladder		spleen		thymus		ovaries/testes		eye		kidney		heart		lung		lymph node		brain		intestine			
***Nkrp1a***																															
B6			-a		–		–		–		+		+		+		–		–		–+		+		++		–		–		
129			–		–		–		–		+		+		++		–		–		–		+		++		–		–		
BALB			–		–		−/+		–		−/+		−/+		+		–		–		–		++		+		–		–		
microarray^b^			–		–		–		–		+		–		–		–		–		–		–		+		–		–		
***Nkrp1c***																															
B6			–		–		+		−/+		+++		++		−/+		−		−/+		-		++		++		–		+		
129			–		–		+		−/+		++		++		−/+		−/+		−/+		−/+		+		++		–		+		
BALB			–		–		++		−/+		+		+		+		−		−/+		+		++		+		–		+		
microarray			+		–		+		–		+		+		–		–		–		–		+		+		–		–		
Nkrp1d/b																															
B6			+		−/+		+		+		++		++		+		−/+		+		+		++		++		-		++		
129			+		–		–		−/+		+		+		+		−/+		–		+		++		++		–		+		
BALB			+		–		+		+		+		+		−/+		−/+		–		+		++		+		–		+		
microarray			+		+		+		+		+		+		–		+		+		+		+		+		–		+		
Nkrp1f																															
B6			–		–		+		–		++		+		–		–		–		–		++		++		–		+		
129			–		–		+		–		+		+		–		–		–		–		+		+++		-		+		
BALB			–		–		++		–		+		+		–		–		–		–		++		++		–		+		
microarray			–		–		–		–		+		–		–		–		–		–		–		+		–		+		
Nkrp1g																															
B6			–		–		–		–		+		+		–		–		–		–		+		−/+		–		+		
129			–		–		–		–		+		+		+		–		–		–		–		−/+		−/+		+		
BALB			–		–		–		–		+		+		+		–		–		–		–		–		–		+		
microarray		ND^c^																													

a -, no expression; −/+, very weak expression; +, weak to moderate expression; ++, strong expression; +++, very strong expression.

b Microarray data were obtained from the Mouse MOE430 Gene Atlas database at BioGPS portal server in order to compare with results of RT-PCR.

c ND, not determined.

**Figure 6:**
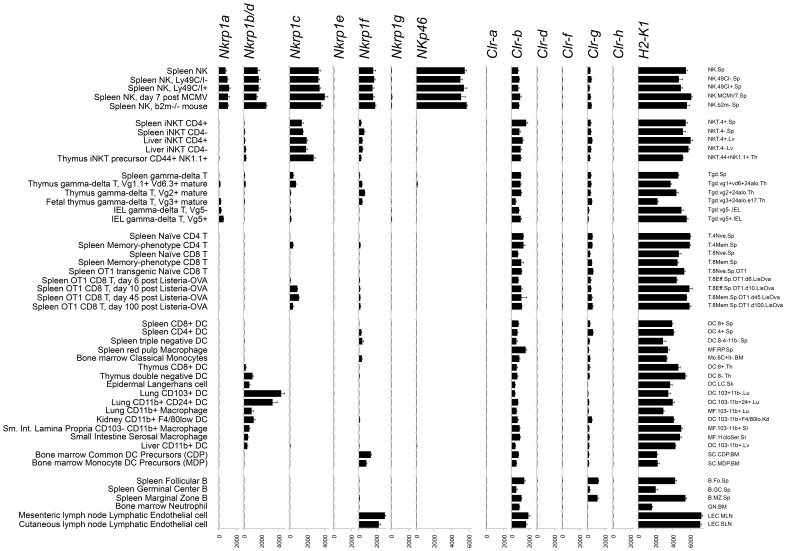
Expression of *Nkrp1* and *Clr* genes in immune cell types revealed by transcriptome micro-array. Normalized hybridization is shown ± SEM. Text at the left indicates brief subset descriptions, while text at the right indicates Immunological Genome Project cell population names. Data was obtained from the Immunological Genome Project (NCBI GEO Series GSE15907) [34]. iNKT, invariant NKT cell; IEL, intra-epithelial lymphocyte; DC, dendritic cell; MCMV, murine cytomegalovirus.

Micro-array data comparing many immune cell types is also publicly available as part of the Immunological Genome Project [34]. Expression of *Nkrp1* and *Clr* genes in selected populations is depicted in **Figure 6**. As previously observed, *Nkrp1a*, *Nkrp1b/d*, *Nkrp1c*, and *Nkrp1f* are expressed by NK cells, while *Nkrp1c* is additionally expressed in NKT cells and some activated/memory T cells. γδ T cells show some signal for several *Nkrp1* receptors, but subsets differ in the predominant receptor expressed. Interestingly, several populations isolated to be tissue dendritic cells or macrophages show substantial expression of *Nkrp1b/d*. The markers used to sort these cells (including MHC-II^+^, CD11b^+^, CD11c^+^) can also be found on NK cells under certain conditions [35], [36]. However, these *Nkrp1b/d* ^+^ populations lacked expression of the classical NK cell markers *Nkrp1c* (NK1.1 in B6 mice) and *Ncr1* (NKp46). This implies that lung, kidney, skin, thymus, and small intestine may contain either: a) *Nkrp1b/d* ^+^ DC and macrophages or b) *Nkrp1b/d* ^+^ *Nkrp1c* ^-^ *Ncr1* ^-^ non-conventional NK or lymphoid cells that co-purify with DC and/or macrophages in these isolations. Additionally, *Nkrp1f* transcripts were detected in bone marrow DC/monocyte precursors and lymph node endothelial cells (**Figure 6**). Within the *Clr* family, widespread *Clr-b* expression was confirmed in many hematopoietic cell types with levels correlated with MHC-I. *Clr-g* was also detected in many populations, but no substantial signal was detected for *Clr-a, Clr-d, Clr-f,* and *Clr-h* in any hematopoietic cell types tested.

### Identification of Specific Cell Types Expressing *Clr* Genes

**Figure 7:**
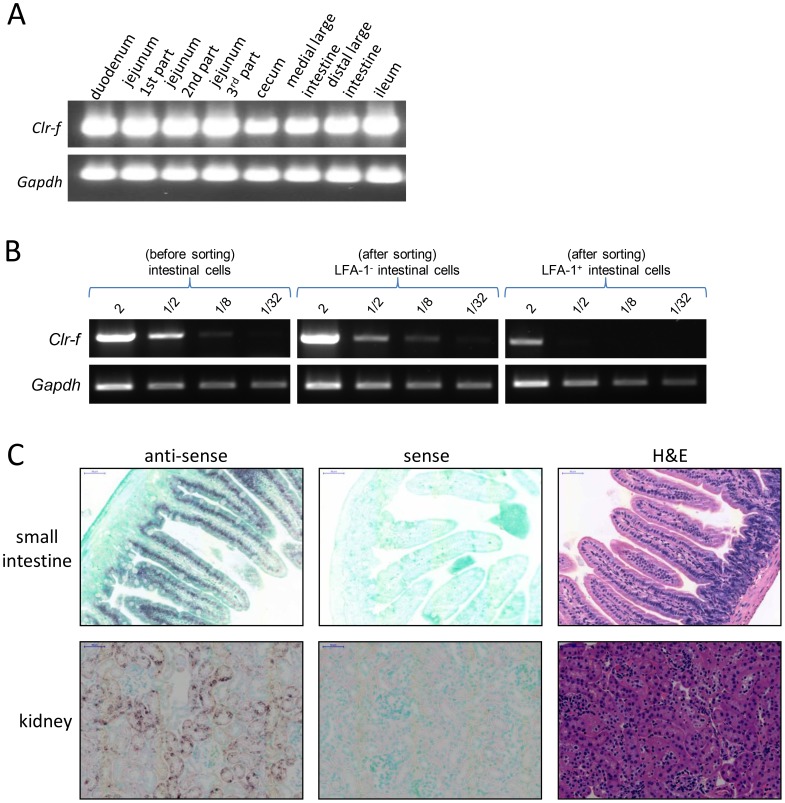
*Clr-f* is expressed in intestinal epithelial cells. (A) Different sections of the small and large intestine from a PBS-perfused B6 mouse were separated and processed for RNA isolation. RT-PCR was performed on the resulting cDNAs with *Clr-f*-specific and *Gapdh*-specific primers. (B) Enriched *Clr-f* expression in non-lymphocytes of the intestine. A section of the small intestine was collagenase-digested and a single-cell suspension was prepared and stained with anti-LFA-1 mAb followed by sorting into lymphocyte and non-lymphocyte (epithelial) populations from which RNA was isolated and used for semi-quantitative *Clr-f* RT-PCR. The amount of cDNA in micrograms used in each reaction is indicated. (C) In situ hybridization reveals that *Clr-f* is abundantly expressed in intestinal epithelial cells and kidney tubular epithelium. DIG-labeled sense (control) and anti-sense RNA probes were used for hybridization to paraffin-embedded intestinal (jejunum) and kidney sections. Hybridization location of the probes was revealed by an alkaline-phosphatase-conjugated anti-DIG secondary followed by (brown) color substrate development. H&E staining of different section of the same organ reveals cellular organization of the tissue. The scale bars in the intestine and kidney sections indicate a length of 50 µm.

The high-level expression of *Clr-f* mRNA in the intestine is in agreement with previous studies [4], and prompted us to attempt to identify the specific cell types expressing this gene. Interestingly, *Clr-f* was expressed in all tissue sections of the small and large intestine (**Figure 7A**), as detected by RT-PCR. Therefore, enzymatic separation of intestinal cells followed by sorting into epithelial (non-hematopoietic) and leukocyte (hematopoietic) subsets based on the expression of the LFA-1 integrin marker (CD11a/CD18) was performed, and documented that *Clr-f* transcripts were mainly expressed within the LFA-1^–^ epithelial (non-hematopoietic) cells (**Figure 7B**). To confirm the specific cell type in the intestine expressing *Clr-f* transcripts, *in situ* RNA hybridization was employed using a *Clr-f* anti-sense RNA probe. This method was selected because mAb are not generally or commercially available for most members of the Clr family. The anti-sense *Clr-f* probe clearly and specifically hybridized to the nuclei of the epithelial cells lining the villi of the small intestine (**Figure 7C**). Importantly, no staining was detected in the lamina propria, which contains resident lymphoid cells. *In situ* RNA hybridization results also revealed that the previous detection of *Clr-f* transcripts by RT-PCR in the kidney was likely due to expression by kidney tubular epithelial cells (**Figure 7C**).

**Figure 8:**
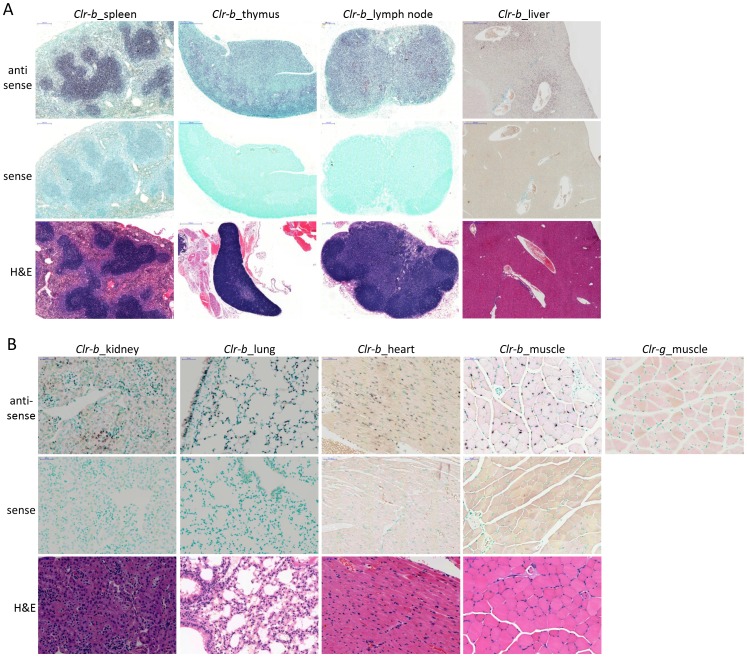
Wide-spread expression of *Clr-b* in lymphoid and non-lymphoid tissues and cell types. In situ hybridization was performed on tissue sections from a PBS-perfused B6 mouse. Hybridization of the indicated (A) primary and secondary lymphoid organs and (B) non-lymphoid organs was performed with a DIG-labeled anti-sense *Clr-b* RNA probe and revealed with an alkaline phosphatase-conjugated anti-DIG secondary mAb. Control in situ hybridization with a DIG-labeled *Clr-b* sense RNA probe is shown. Anti-sense *Clr-g* staining of hind leg muscle tissue is provided as a hybridization specificity control. H&E staining is provided to identify cell types and reveal organ structure. The scale bars indicate 200 µm in the lymph node and spleen, 500 µm in the liver and thymus, and 50 µm in the kidney, lung, heart, and muscle.

**Figure 9:**
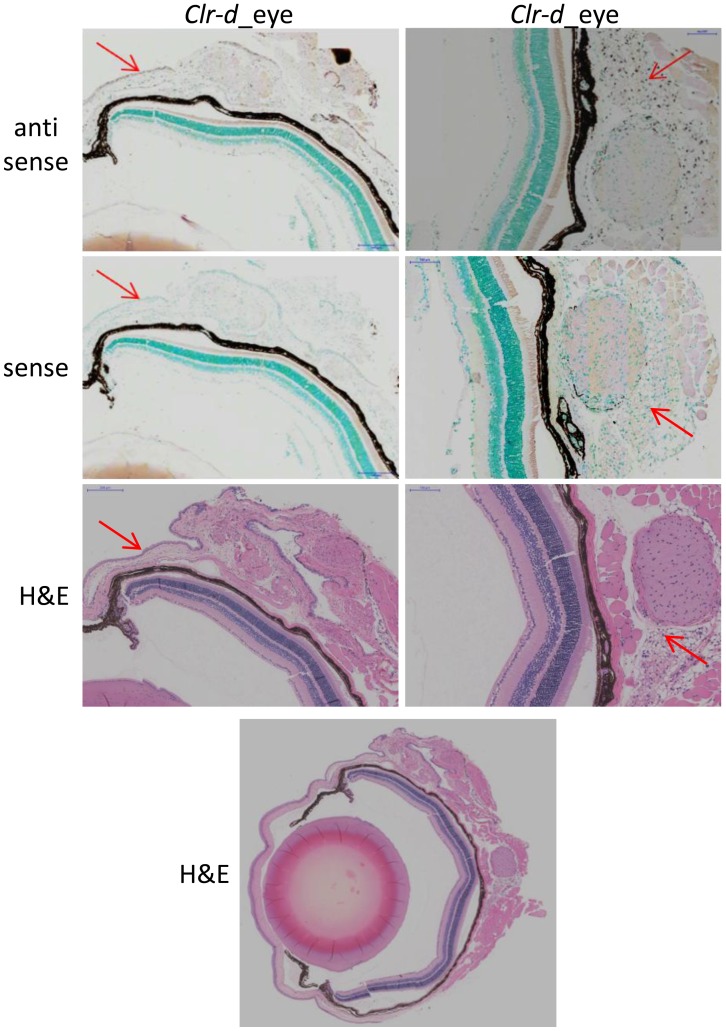
*Clr-d* is expressed in distinct areas of the mouse eye. A DIG-labeled RNA probe for *Clr-d* was used to identify regions of the eye expressing this gene. Expression was detected in the corneal epithelial progenitor cells (left column) and the cell mass surrounding the optic nerve (right column). Red arrows indicate areas of positive staining. Sense (control) RNA hybridizations are shown along with H&E stains to elucidate eye cell types and architecture. For organ orientation an H&E stain of the whole eye is provided at the bottom. The scale bar in the sclera epithelial cell section indicates 200 µm, and 100 µm in the optic nerve section.

**Figure 10:**
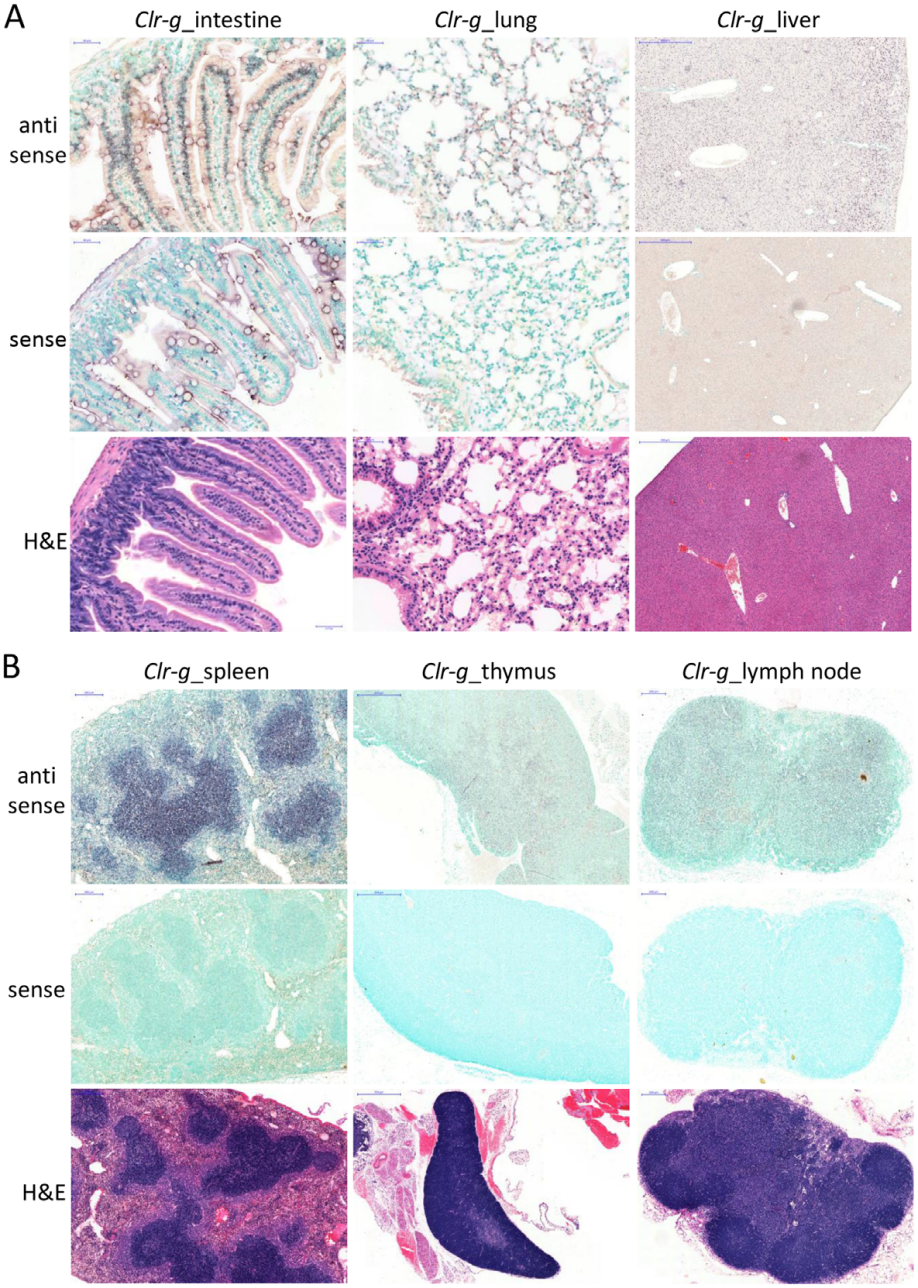
Lymphoid and non-lymphoid cell expression of *Clr-g*. Paraffin-embedded tissue sections of the (A) small intestine (jejunum), lung, and liver, and (B) spleen, thymus, and inguinal lymph node were prepared from a PBS-perfused B6 mouse followed by hybridization with DIG-labeled sense (control) or anti-sense RNA probes. Hybridization was visualized with an alkaline phosphatase-conjugated anti-DIG mAb followed by a (brown) color substrate. An H&E stain of a subsequent tissue section is provided to visualize organ architecture. The scale bars of each tissue section are as indicated for the previous figures.

*In situ* RNA hybridizations were subsequently conducted to identify the specific cell types expressing the remaining *Clr* transcripts in tissues that were documented to be positive by RT-PCR and microarray. The results are collectively as follows:

- ***Clr-b.*** As shown in **Figure 8A**, *Clr-b* transcripts are detected at high levels in splenic lymphoid follicle (B cell zone) cells and less obviously in the periarteriolar lymphoid sheath (T cell zone). *Clr-b* transcripts are also abundantly present in the thymus and in lymph node tissues, in agreement with previous cell-type specific flow cytometric analyses [13]. In the liver, *Clr-b* is expressed highly and specifically around the central vein area, portal triad area, and hepatocytes close to the epithelial stroma, with weaker expression in other areas as well. Non-lymphoid organ *Clr-b* hybridizations are shown in **Figure 8B**. Like *Clr-f*, *Clr-b* transcripts were detected in the kidney. However, unlike *Clr-f*, kidney *Clr-b* staining was restricted to the glomerular and interstitial areas in a pattern indicative of capillary endothelial cells. *Clr-b* was detected in bronchial epithelial and endothelial cells, as well as endothelial cells of the lung parenchyma. Surprisingly, myocyte nuclei of skeletal muscle and heart tissue are also clearly stained with the *Clr-b* probe. As a specificity control, muscle section hybridization was also carried out using a *Clr-g* probe and found to be negative, in agreement with RT-PCR and microarray results. Our results are consistent with previous reports documenting *Clr-b* expression by *in situ* RNA hybridization in lung, heart, small intestine, skeletal muscle, and spleen [31], although the specific cell types involved were not identified in this report.
- ***Clr-d*** **.** The antisense *Clr-d* probe detected expression by different cell types in the eye, specifically the epithelial layer of the sclera (the outermost cells; **Figure 9**, left panel) and the choroid body, which is the large mass of cells surrounding the optic nerve (**Figure 9**, right panel). The latter are possibly glial cells (astrocytes or oligodendrocytes), but could also be residual blood cells (after PBS perfusion) circulating in the tissue or endothelial cells from blood vessels. Low levels of *Clr-d* transcripts could also be detected in the corneal epithelium (data not shown).
- ***Clr-f*** **.** As described above, *Clr-f* was detected specifically in intestinal epithelial cells and kidney tubular epithelial cells (**Figure 7C**).
- ***Clr-g.*** *Clr-g* transcripts were found to be present in intestinal epithelial cells, although at apparently lower levels than *Clr-f*, as well as an unidentified cell subset within the lamina propria (**Figure 10A**). Interestingly, *Clr-g* appears to be transcribed in bronchial epithelial cells, endothelial cells, and alveolar epithelial cells of the lung (**Figure 10A**), but to a lesser extent than *Clr-b*. Although the lung epithelial cells are clearly stained in the bronchi, the expression is sporadic. The lung endothelial cells also show more diffuse expression, as well as alveolar epithelial cells (pneumocytes), specifically type-2 alveolar cells, which are large surfactant-producing cells. Similar to *Clr-b*, *Clr-g* transcripts were also detectable in the B cell areas of the spleen, and the majority of the thymus and lymph node tissues, but the latter two organs appear to express lower levels of *Clr-g* compared to *Clr-b* (**Figure 10B**). Like *Clr-b*, *Clr-g* transcripts were also detected in the liver (**Figure 10A**), despite RT-PCR and microarray both being negative. However *Clr-g* was expressed more evenly throughout the liver than *Clr-b*. The reason for the *in situ* hybridization versus RT-PCR/microarray discrepancy may be due to blood cells that were removed for the latter analyses.

## Discussion

The observation that the *Nkrp1* and *Ly49* genes are closely linked within mouse chromosome 6 led to the designation of this region as the NK gene complex, or NKC [37]. Although related, the two receptor families encoded by these genes have undergone different evolutionary strategies for their contribution to the innate immune system and expression by NK cells. Firstly, the ligands for the two receptor families are unrelated, with the Ly49 receptors recognizing type-I-transmembrane, Ig-superfamily-related MHC-I molecules, while the NKR-P1 receptors recognize type-II-transmembrane, C-type lectin-related Clr molecules. Secondly, the genes encoding the Clr (*Clec2*) family are intermingled among the *Nkrp1* (*Klrb1*) genes, while the genes encoding the MHC and Ly49 receptors are found on separate chromosomes, with concomitant ramifications for the strength and number of possible self-specific receptors that are responsible, in part, for NK cell responsiveness [38], [39]. Thirdly, the *Ly49* genes are highly polymorphic at the allelic level, as well as highly elastic at the haplotype level, with a known structural gene content variation of between 8–22 genes documented to date per cluster [30], [40]. In contrast, as shown in the present and previous studies [6], [7], the *Nkrp1/Klrb1* (and *Clr/Clec2*) gene number appears to be stable in at least three different inbred mouse strains, with minimal but focused allelic variation. It is possible that while the B6, 129, and BALB/c strains all represent a single categorical *Nkrp1* haplotype, there may be other mouse strains possessing alternate *Nkrp1* clusters with different gene numbers. In limited support of this, aCGH and Southern analyses of a large panel of mouse strains suggest that there may be at least four distinct *Nkrp1* haplotypes (compared to five *Ly49* haplotypes), and that the B6 and 129/BALB haplotypes may broadly characterize two different groups [24], although this study concluded that *Nkrp1* group haplotype diversity is significantly less than that displayed by *Ly49*. The *Nkrp1* and *Clr* genes are highly conserved, as suggested by comparison of the Southern blot analyses of the *Clr/Clec2* family genomic RFLP patterns reported here (**Figure 4**), and by *Nkrp1/Klrb1* Southern blot analyses reported previously [24]. Finally, whereas the ligands for the Ly49 receptors are expressed on almost all nucleated cell types, thus excluding mature erythrocytes and thrombocytes, we find that the ligands for the NKR-P1 receptors appear to be expressed in a tissue- and organ-restricted manner, perhaps with the exclusion of Clr-b (and possibly Clr-g). Specifically, we observed isoform/locus-specific Clr expression not only among hematopoietic cells (leukocytes), but also among non-hematopoietic epithelial cells (intestine, kidney, eye), endothelial cells (lung, kidney), myocytes (hind leg muscle, heart), and possibly neuronal cell types (eye). Thus, while Ly49/MHC-I immunosurveillance is global, the NKR-P1/Clr system appears to be more tissue-specific.

### Role of Nucleotide Repeat Elements in *Nkrp1/Ly49* Evolution

We had previously hypothesized that the high percentage of LINE-1 elements in the *Ly49* cluster, calculated to be double the genomic average, contributes to the rapid contraction and expansion of this region by acting as ‘sliding’ regions of homology during meiotic sister chromatid cross-overs [3]. LINE-1 elements are endogenous retro-elements that contain promoters and frequently code for functional reverse transcriptase and/or endonuclease activities, but don’t contain additional *gag*-*pro*-*env* gene products like standard retroviruses. Since the *Nkrp1/Clr* cluster appears to be relatively stable, as concluded by the present sequencing and previous BAC contig and aCGH analyses of multiple inbred strains [6], [7], [24], it can serve as a natural test of this hypothesis. Indeed, it was observed that the LINE-1 make-up of the *Nkrp1/Clr* region was close to the genomic average. However, an unexpected finding was the high percentage of LTR retro-elements in the *Nkrp1/Clr* cluster, which was approximately twice the genomic average, as well as twice the percentage observed within the sequenced *Ly49* clusters (**Table 3**) [29]. LTR retro-elements (or endogenous retroviruses) are the result of germ-line infection and transmission of *bona fide* retroviruses (with LTR regions and coding regions for *gag*, *pro*, *pol*, *env*). There are few to no LTR retro-elements still capable of replication in humans (called HERVs), as they appear to be heavily mutated or contain deletions [41]. In contrast, mice have hundreds of functional LTR elements (e.g., MusD and IAP) that appear to be still actively translocating within the mouse genome [41]. Despite being largely non-infectious, human cells can still express gene products derived from these LTR retro-elements that can assemble in virus-like particles upon activation. Indeed, some autoimmune diseases have been suspected to be linked to these retro-element activities [42]. The reason for the over-representation of LTR sequences in the *Nkrp1/Clr* region is unclear at present.

### *Clr* Expression in Non-hematopoietic Cells

The expression of several *Clr* family members in non-hematopoietic cells, specifically within various kinds of tissue-specific epithelial cells, myocytes, endothelial cells, and possibly neurons, suggests that NKR-P1 receptor regulation of NK cell function may in fact be tailored for immunosurveillance of many if not most organs and tissues. This may represent a paradigm shift for NK cell biology, as KIR/Ly49 regulation via MHC-I expression appears to be more global, at least for most nucleated cells. Various *Clr* transcripts were detected by RT-PCR and *in situ* RNA hybridization in non-hematopoietic cells, including epithelial cells of the kidney, intestine, lung, and eye. *Clr* transcript expression was also detected in myocytes of the heart and hind leg muscle. Contaminating leukocytes may account for some of the signal by RT-PCR analysis, but *in situ* RNA hybridization of tissue sections may be used to visually exclude blood cells. It is possible that the antisense RNA probe hybridization detected in the various organs/tissues is not due to annealing with a functional transcript; for example, hybridization could result from binding to non-functional alternatively spliced transcripts or RNA molecules with coincidental complementarity. However, full-length sequence-verified cDNAs were amplified and cloned from multiple tissues (which were PBS perfused to eliminate blood cells), thus supporting the finding that the tissue-specific expression of the genes appears to coincide with detectable *in situ* RNA hybridization within specific cell types. Additionally, while intact transcripts may be detectable, it is also possible that the corresponding protein may not be properly translated or expressed at the cell surface until the appropriate cellular stimuli are provided. This possibility awaits the generation of mAb specific for the remaining Clr family members. However, it remains likely that the cellular transcripts detected in the present study are translated at least in part.

### Species Conservation of *Clr* Expression and Function

The *Clr/Clec2* loci have also been documented to be part of a multi-gene family in the rat. The *Clr/Clec2* gene closest to the *Cd69* locus and the *Ly49* cluster is *Clec2d11/Clr11* in the rat and *Clec2d/Clr-b* in the mouse. These two genes are clearly homologous and likely orthologous, but also most likely function similarly since rat *Clec2d11/Clr11* expression has been broadly documented in the spleen, thymus, liver, heart, muscle, and kidney, similar to mouse *Clec2d/Clr-b* [43]. The recognition of rClr11 by the inhibitory rNKR-P1B receptor also highlights conservation of function with mClr-b, which is recognized by mNKR-P1B [44], [45]. Rat Clr10 is thought to be orthologous to mClr-a, based on sequence and location, and was previously reported to be highly expressed in the thymus and moderately expressed in the spleen and liver [43]. On the other hand, while we detected mClr-a to be only weakly expressed in the intestine, our RT-PCR results were otherwise in agreement with previous microarray analyses of mClr-a expression. The expression of rClr10 in the intestine remains unknown. The only other somewhat likely orthologous relationship between the mouse and rat systems is mClr-f and rClr9; however, no expression data is currently available on the rat counterpart for functional comparison.

While human LLT1 is thought to be the closest homologue and potential orthologue of mClr-b due to its binding of the inhibitory hNKR-P1A (CD161) receptor [17], [18], the LLT1 expression pattern clearly contrasts with that of mClr-b in that it is more highly restricted in resting leukocytes, with significant inducible expression documented in TLR-activated pDC and B cells, as well as TCR-activated T cells and IL-2-activated NK cells [46], [47]. Mouse Clr-b, on the other hand, appears to be constitutively expressed on leukocytes [13], but may also have evolved additional functions on non-hematopoietic cells. As Clr-b expression is down-regulated upon cell stress due to transformation, DNA damage, or infection, it seems to function as a marker of ‘health’ for NK cells [14], [15], [44]; however, its inducible expression on the above cell populations in response to activation still remains undocumented. It thus remains possible that LLT1 may more closely represent a ligand for the other inhibitory NKR-P1 receptor in the mouse, mNKR-P1G, which in turn may more closely represent a hNKR-P1A homologue. These possibilities await further detailed bioinformatic and functional characterization of the NKR-P1/Clr system.

Likewise, the constitutive expression of Clr-f ligand for the inhibitory NKR-P1G receptor in the intestine and kidney suggests that it may also act as a marker of health in these organs, which may contain NK cells or other lymphocytes expressing NKR-P1G. Clr-f joins ICOS-L and B7-H1 as a probable immune modulator expressed by renal tubular epithelial cells [48]. The tubular epithelium of the kidney and villus epithelium of the small intestine are susceptible to injury from not only bacterial and viral pathogens, but also ischemia-reperfusion injury, oxidative stress, nephrotoxins, inflammation, and immune disorders. We further found constitutive expression of Clr-g in various organs and tissues, suggesting that it also may act as a regulator of cell health for NK cells or other lymphocytes via NKR-P1G. However, Clr-g is also a common ligand for the putative stimulatory NKR-P1F receptor [6], [16]; moreover, it is not yet clear whether NKR-P1F is functionally active in the same way that the NKG2D or Ly49D receptors signal, since NKR-P1F does not appear to induce cytokine production or cytotoxicity by NK cells [9]. Similarly, Clr-d, which we found to be uniquely expressed in the eye, is a common ligand for the NKR-P1F and NKR-P1G receptors [6], but the functional role of receptor-ligand interactions in this organ is unclear.

In summary, sequencing analyses in three different inbred mouse strains confirms previous gene-mapping and aCGH studies, suggesting that the *Nkrp1/Clr* gene cluster is highly conserved compared to the nearby *Ly49* region, especially with respect to gene number stability, which the *Ly49* region lacks. Genome sequencing further indicates a correlation between conservation and specific repetitive element prevalence, suggesting that these elements may also be involved in immune gene evolution, but as a facilitating mechanism rather than immune selection pressure. Complementary expression analysis of transcripts from this cluster suggest that NKR-P1-mediated immunosurveillance may be tissue- and ligand-specific, rather than global as per the Ly49/KIR system in recognition of MHC-I molecules.
